# Assessment and Management of Older Patients With Transthyretin Amyloidosis Cardiomyopathy: Geriatric Cardiology, Frailty Assessment and Beyond

**DOI:** 10.3389/fcvm.2022.863179

**Published:** 2022-05-17

**Authors:** Biobelemoye Irabor, Jacqueline M. McMillan, Nowell M. Fine

**Affiliations:** ^1^Cumming School of Medicine, University of Calgary, Calgary, AB, Canada; ^2^Division of Geriatrics, Departments of Medicine and Community Health Sciences, Calgary, AB, Canada; ^3^Division of Cardiology, Departments of Cardiac Sciences, Medicine and Community Health Sciences, Libin Cardiovascular Institute, Calgary, AB, Canada

**Keywords:** heart failure, geriatrics, frailty, cardiac amyloidosis, transthyretin, therapy

## Abstract

Transthyretin amyloidosis cardiomyopathy (ATTR-CM) is commonly diagnosed in older adults, in particular the wild-type (ATTRwt), which is regarded as an age-related disease. With an aging population and improved diagnostic techniques, the prevalence and incidence of ATTR-CM will continue to increase. With increased availability of mortality reducing ATTR-CM therapies, patients are living longer. The predominant clinical manifestation of ATTR-CM is heart failure, while other cardiovascular manifestations include arrhythmia and aortic stenosis. Given their older age at diagnosis, patients often present with multiple age-related comorbidities, some of which can be exacerbated by ATTR, including neurologic, musculoskeletal, and gastrointestinal problems. Considerations related to older patient care, such as frailty, cognitive decline, polypharmacy, falls/mobility, functional capacity, caregiver support, living environment, quality of life and establishing goals of care are particularly important for many patients with ATTR-CM. Furthermore, the high cost ATTR treatments has increased interest in establishing improved predictors of response to therapy, with assessment of frailty emerging as a potentially important determinant. Multidisciplinary care inclusive of collaboration with geriatric and elder care medicine specialists, and others such as neurology, orthopedic surgery, electrophysiology and transcatheter aortic valve replacement clinics, is now an important component of ATTR-CM management. This review will examine current aspects of the management of older ATTR-CM patients, including shared care with multiple medical specialists, the emerging importance of frailty assessment and other considerations for using ATTR therapies.

## Introduction

Transthyretin amyloidosis cardiomyopathy (ATTR-CM) is caused by the deposition of misfolded transthyretin (TTR) proteins as amyloid fibrils in the myocardial extracellular space (1). TTR is a predominantly hepatic derived transport protein responsible for transporting thyroxin and retinal binding protein (hence the name ‘*trans-thy-retin'*) through the circulation and is also commonly referred to as prealbumin (2). ATTR-CM is subdivided into two types; hereditary (ATTRh, also variably referred to in the literature as mutant or variant type), which is caused by a mutation of the TTR gene, and wild-type ATTR (ATTRwt, previously referred to as senile systemic amyloidosis), which is an age-related disorder more commonly affecting men and occurring in the absence of a TTR gene mutation (3, 4). ATTR-CM is predominantly a disease of older adults. While the true prevalence of ATTR-CM is uncertain, it is commonly considered to be an underrecognized cause of heart failure in the community (4). Improvements in diagnostic approaches, in particular significant advances in non-invasive cardiac imaging assessment, and disease awareness, along with an aging population, have resulted in increasing incidence rates of ATTR-CM (5). This, coupled with the improving availability of approved novel disease modifying therapies that improve survival for ATTR-CM patients are likely to contribute to a steady rise in prevalence in the future as patients live longer (5). As a result of these significant advancements, optimizing the care of older ATTR-CM patients has become increasingly important. This task has implications for both the assessment and management of older ATTR-CM patients and presents a number of unique challenges for clinicians caring for this population. This review will examine several current aspects of the management of older ATTR-CM patients, including shared care with multiple medical specialists, the emerging importance of frailty assessment and other considerations for using ATTR therapies.

## Epidemiology

While multiple studies have attempted to estimate the prevalence of ATTR-CM, the true prevalence remains uncertain. A recent community cohort study of patients over the age of 60-years with heart failure and preserved ejection fraction (EF) and increased left ventricular (LV) wall thickness found an ATTR-CM prevalence of 1.3% of patients clinically diagnosed, and 6.3% among patients who prospectively underwent dedicated screening for ATTR-CM (6). The expanded use of technetium nuclear scintigraphy with bone-seeking radiotracer as part of the diagnostic approach for non-invasively identifying ATTR-CM has significantly improved detection rates (7, 8). Several recent studies have demonstrated the prevalence of ATTR-CM within different clinical populations of patients with concurrent cardiovascular disease presenting to referral centers. For example, 13% of subjects over 60 years-of age hospitalized for a diagnosis of heart failure with preserved ejection fraction (HFpEF) and increased LV wall thickness were found to have ATTRwt (9), while 16% of patients referred for transcatheter aortic valve replacement (TAVR) also had ATTR-CM (10), with the mean age of those respective cohorts being 82 and 84 years.

While in endemic regions ATTRh is the predominant ATTR-CM subtype, in most other areas ATTRwt is regarded as more common (11). The median age of ATTRwt diagnosis is >70 years-of-age (8). ATTRh has a more variable demographic profile, which is significantly influenced by genotype (11). The presence of cardiac involvement of ATTRwt was previously often detected on postmortem examination and considered to be part of the normal aging process (12). Improved histopathological techniques subsequently demonstrated in one autopsy series that approximately 25% of all subjects ≥80 years-of-age has ATTRwt cardiac infiltration, although the rate of penetrance of clinical disease was uncertain (12).

While some TTR gene mutations can occur in younger patients (early onset variants) and typically cause transthyretin amyloidosis polyneuropathy (also referred to as familial amyloidosis polyneuropathy, FAP), namely the pV50M mutation, many other genotypes, particularly those more commonly associated with ATTR-CM, often occur in older patients. These include the pV142I mutation, which occurs in patients of African and Caribbean descent and has an estimated prevalence of 4% in the United States (US), although the clinical penetrance in this population is uncertain, and the pT80A mutation, occurring mostly in patient of Irish decent (11). A late onset form of the pV50M mutation can also cause a predominantly cardiac phenotype of ATTR. The Transthyretin Amyloidosis Outcome Survey (THAOS), a large multicenter international longitudinal observational registry of patients with ATTR, reported a mean age of all ATTR-CM patients of 70 years, with a mean age of 69 years for US patients with pV142I mutation (11). In the US, ATTRwt made up 48% of cases while pV142I was the dominant ATTRh mutation and accounted for 23%. Outside the US, patients with pV50M mutation accounted for 76% of all patients with ATTR and 28% of patients with ATTR-CM, while ATTRwt made up 5 and 26%, respectively (11).

## Clinical Manifestations of ATTR in Older Patients

**Figure 1:**
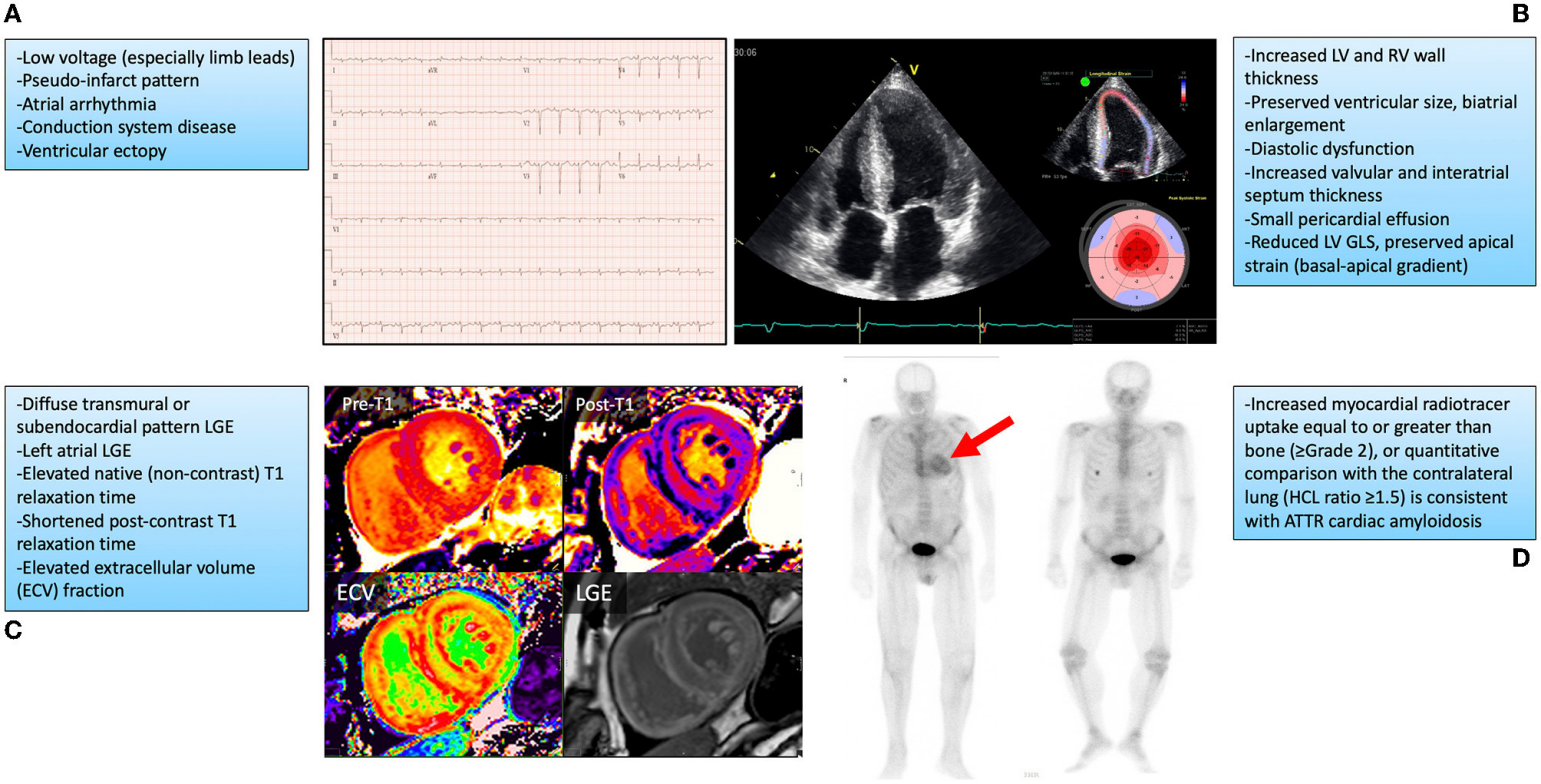
Findings on cardiovascular investigations associated with cardiac amyloidosis along with representative examples. **(A)** Typical electrocardiogram findings and representative example. **(B)** Echocardiogram findings, with imaging from the apical 4-chamber view (left image) showing biventricular wall thickening, preserved ventricular size, valve thickening, and biatrial enlargement, with longitudinal strain measurement on speckle-tracking echocardiography (top right) showing preserved apical strain with impaired basal and middle segment values (bottom right). **(C)** Cardiovascular magnetic resonance imaging findings with representative examples showing diffuse elevation in native T1 (no contrast; Pre-T1) mapping (top left), reduction in post contrast T1 (Post-T1) mapping (top right), increased extracellular volume (ECV; bottom left), and subendocardial late gadolinium enhancement (LGE) of the left and right ventricles (bottom right). **(D)** 99 mTc-pyrophosphate nuclear scintigraphy showing increased myocardial uptake in a patient with transthyretin cardiac amyloidosis (red arrow, left panel) and absent myocardial uptake in a patient without this diagnosis (right panel). GLS, global longitudinal strain; HCL, heart/contralateral lung ratio; LV, left ventricular; RV, right ventricular. Fine et al., (4).

Although cardiac manifestations may predominate for many patients, ATTR is a multisystem disease that includes somatic and autonomic neurologic, musculoskeletal, gastrointestinal and ophthalmologic symptoms, among others (2, 13, 14). Many disease manifestations may mimic or exacerbate other age-related disorders, which can make clinical recognition difficult in older patients, particular in the early stages. Heart failure remains the most common clinical presentation for ATTR-CM patients (2). In many patients this is slowly progressive and initially manifests as exercise intolerance and worsening dyspnea on exertion, advancing to overt biventricular heart failure symptoms, including right heart failure causing peripheral edema, fatigue, early satiety and abdominal bloating, and left heart failure causing pulmonary congestion, orthopnea and paroxysmal nocturnal dyspnea. In early stages, older patients may not seek medical attention and attribute their symptoms to aging. As symptoms progress and patients seek medical attention, clinicians may not recognize ATTR-CM, either because of limited awareness or a lack of other typical ‘red-flag' findings associated with more advanced stages of this disease (15). Patients may initially be mis-diagnosed with HFpEF, a common disorder that represents approximately half of all heart failure in the community (16, 17). ATTR-CM predominantly presents with preserved LV EF (although EF can become reduced in later stages), making differentiation of these two diseases difficult (4). Phenotypic similarities between ATTR-CM and HFpEF present on cardiac imaging assessment may also include increased LV wall thickness, diastolic dysfunction, and atrial enlargement. Differentiating features may include the presence of increased right ventricular wall thickness which would be more associated with ATTR-CM, along with findings more specific to ATTR-CM by advanced imaging techniques such as: reduced global but preserved apical LV longitudinal systolic strain by speckle-tracking echocardiography, diffuse subendocardial or transmural late-gadolinium enhancement and increased native T1-mapping time and post-contrast extracellular volume quantification using cardiac magnetic resonance imaging (Figure 1) (8, 18–22).

Another cardiovascular manifestation of ATTR-CM that is also commonly associated with aging are rhythm disturbances, in particular atrial fibrillation and conduction system disease. The incidence of rhythm disturbances has been reported to be higher in ATTRwt compared with patients with ATTRh due to pV142I mutation (65.5 vs. 32%) (11). Recent reports have estimated the prevalence of atrial fibrillation among ATTR-CM patients to be 44–70% (23–25). It is uncertain whether atrial fibrillation is caused by structural cardiac changes associated with ATTR-CM (such as elevated LV filling pressure causing atrial enlargement), or is intrinsic to the disease, however ATTR amyloid is known to infiltrate the atria (26). Conduction system disease is an electrophysiologic complication of ATTR-CM that can also be associated with aging. The need for permanent pacemaker implantation due to high grade atrioventricular block and/or symptomatic bradycardia is a recognized disease complication and is associated with a worse prognosis in ATTRwt patients, although the incidence of pacemaker implantation in ATTR-CM patients reported in the literature is highly variable (23, 27). Lastly, an important cardiovascular complication of ATTR-CM that can also be associated with aging is aortic stenosis (2, 10, 14, 28, 29). Aortic stenosis is more commonly described in ATTRwt, although can occur in ATTRh patients. ATTR-CM patients who present with severe aortic stenosis may initially be referred for consideration of TAVR due to their increased age and higher risk for surgical aortic valve replacement, and therefore recent efforts to screen patients for ATTR-CM have focused on those referred for TAVR (10).

Peripheral neuropathy is a hallmark feature of some ATTRh genotypes, notably the pV50M mutation (30). Recently it has been recognized that ATTRwt patients can also develop peripheral neuropathy (31, 32). Similar to cardiovascular manifestations, symptoms of peripheral neuropathy can also be associated with other more common conditions that increase in prevalence with age, such as diabetes, thyroid dysfunction, monoclonal gammopathy and vitamin deficiencies, making clinical recognition of ATTR challenging, particularly in early stages of disease (33). Carpal tunnel syndrome is also very common among ATTR patients with both subtypes and can present well in advance of cardiomyopathy, in some patient years in advance (15). Carpal tunnel syndrome is also very common in the community and associated with a wide range of pathologies. Carpal tunnel syndrome can cause numbness, pain, and loss of fine motor skills in the hands and fingers. Nakagawa et al. found a high prevalence of carpal tunnel syndrome as an initial symptom of ATTRwt in 55% of subjects, followed by heart failure symptoms in 45% (34). Other musculoskeletal disorders, such as biceps tendon rupture, spinal stenosis, and orthopedic problems such as joint degeneration are further examples of conditions associated with aging that are highly prevalent in ATTR patients (2, 13, 35). Autonomic dysfunction is also associated with ATTR and can present with multiple clinical manifestations (5). Important among these are orthostatic hypotension, which can manifest as presyncope or syncope and can exacerbate the risk of falls in older patients. Progressive decline in blood pressure, particularly for patients with a prior diagnosis of hypertension who now require reduction or discontinuation of antihypertensive therapy, is recognized as one of the ‘red flags' for identifying ATTR-CM (15). Other manifestations of autonomic dysfunction include gastrointestinal symptoms such as early satiety, nausea and bloating caused by gastrointestinal dysmotility (which can be exacerbated by heart failure-induced gastrointestinal congestion), along with urinary, bowel and sexual dysfunction. Autonomic dysfunction is associated with a worse prognosis for ATTRwt (36). Vitreous opacities causing blurred vision occur mostly with certain ATTRh genotypes (4, 32). Lastly, hearing loss is common in ATTRwt (37), which may promote social isolation and depression and contribute to frailty in older patients (38, 39).

Perhaps one of the most debilitating and also challenging symptoms to quantify for patients with ATTR-CM that can also be associated with aging is fatigue. Whether a result of other disease manifestations or intrinsically caused by ATTR, fatigue can cause significant limitations for older patients. A summary of ATTR-CM disease manifestations that may also be associated with age-related disorders in provided in Table 1. All of the disease manifestations described above can impair mobility and overall functional status in older ATTR-CM patients, diminishing independence and reducing quality of life. While specialists who typically manage ATTR such as cardiologists or neurologists may effectively manage system-specific symptoms, the global impacts of disease may be more challenging for these clinicians to manage, and geriatricians and elder care specialists may provide complementary assessment and management skills to optimize care in this complex population. Table 2 provides referral indications for geriatric assessment categorized by urgency, while Table 3 describes areas of assessment and potential management strategies utilized by geriatricians.

**Table 1:** Clinical manifestations of ATTR significant for older adults.

**System**	**Manifestations**	**Limitations**
Cardiovascular	-Heart failure -Tachyarrhythmia -Conduction system disease -Aortic stenosis	-Exercise intolerance -Chest pain / dyspnea with exertion -Presyncope, syncope
Neurologic	-Sensorimotor polyneuropathy -Spinal stenosis -Carpal tunnel syndrome	-Poor balance, loss of sensation (numbness and tingling) and muscle strength / weakness -Impaired mobility, reliance on mobility aids -Impaired fine motor skills, loss of ability to perform IADLs and ADLs -Loss of independence
Autonomic	-Orthostatic hypotension -Urinary dysfunction -Early satiety -Bowel habit alteration (constipation, diarrhea) -Sexual dysfunction	-Impaired mobility, increased fall risk -Incontinence of urine and stool -Social isolation, depression
Gastrointestinal	-Early satiety -Malabsorption	-Malnutrition, weight loss
Musculoskeletal	-Generalized weakness and fatigue -Biceps tendon rupture -Degenerative joint disorders	-Pain -Impaired mobility, reliance on mobility aids -Loss of ability to perform IADLs and ADLs -Loss of independence
Visual	-Vitreous opacities	-Visual impairment -Loss of independence
Auditory	-Full frequency hearing loss	-Impaired ability to communicate -Loss of independence -Social isolation, depression

*ADL, activities of daily living; IADL, instrumental activities of daily living*.

**Table 2:** Geriatric referral indications and triage urgency.

**Triage level**	**Timeline**	**Indications**
Emergent	Straight to ED and a same day assessment	acute confusion (delirium), disruptive behavior in the setting of dementia, new onset immobility
Urgent	Seen in clinic within 2 weeks	recent or subacute decline in function, multiple falls in a short period of time, rapid decline in cognition
Routine	>2 weeks	advice on dementia diagnosis or management, complex chronic disease management, decline in functional status, frailty, frequent falls, review of complex medical issues

*ED, Emergency department*.

**Table 3:** Areas of assessment included in a geriatric evaluation and approaches to management.

**Area of assessment**	**Approach to assessment**	**Management**
General	-Obtain the medical history with a focus on conditions common to older adults -Obtain collateral history when appropriate and with consent -Focus on physical health, function, cognitive and affective health - Comprehensive review of both basic and instrumental activities of daily living -Review of social, psychological and environmental determinants of health	-Approach is multidisciplinary and includes allied health involvement including nursing staff, pharmacists, social work, physical and occupational therapy -Due to multifactorial nature of problems in geriatrics, treatment is usually directed at the underlying causes -All treatment recommendations ideally will take into consideration individual patient abilities, preferences and goals
Cognition	-Thorough history from both the patient as well as a family member or friend -Comprehensive physical examination with a focus on the neurological examination, including mental status, as well as objective cognitive testing -Consideration of factors that influence cognition and cognitive testing including age, education and sociocultural and linguistic background -Investigations including blood work and imaging when indicated	-Home care support -Social work support -Referral to day programs -Referral to Alzheimer's Society -Medical therapy when indicated
Depression and anxiety	-Comprehensive history and physical examination -Investigations as needed to rule out other conditions which may mimic depression and/or anxiety -Assessment of patient safety and social supports available	-Referral to counseling and psychiatry services as indicated -Consideration of antidepressant and/or, anxiolytic with consideration of pharmacodynamics and pharmacokinetic changes associated with aging
Malnutrition and weight loss	-Comprehensive history and physical exam to elucidate concerns related to reduced intake, increased energy demands, reduced absorption and/or impaired motility -Screen for food insecurity including a safe, accessible and affordable food supply -Screen for depression -Evaluation for red flags related to malnutrition and weight loss which way may warrant targeted investigations	-Referral to dietician when indicated -Referral to Gastroenterology when indicated -Home care, formal/paid care and family supports -Further investigations when indicated based on the history and physical exam
Urinary incontinence and constipation	-Comprehensive history and physical examination -Review of mobility (ability to access toilet), cognition (ability to recognize need to toilet) -Medication and dietary review searching for contributing factors -Cognitive evaluation as needed -Assessment for lower urinary tract symptoms in men -Evaluation for co-existing neurological symptoms	-Scheduled, prompted and assisted toileting where mobility and cognition are deemed to be contributors -Nonpharmacological and pharmacological management targeted to culprit conditions -Urology/gynecology/ Gastroenterologist evaluation as needed -Urodynamic studies, cystoscopy, endoscopy/ colonoscopy, prostate evaluation (PSA) as indicated -Deprescribing of culprit medications
Balance/gait and falls	-Comprehensive history of impaired gait and falls -Comprehensive physical exam and gait evaluation -Bone health assessment -Laboratory investigations, imaging and further testing (i.e., nerve conduction studies) where indicated	-Physiotherapy -Occupational therapy-mobility aids, reducing environmental fall risks in the home -vitamin D supplementation and encouragement of calcium from the diet (or supplement) -Osteoporosis treatment (e.g., bisphosphonates) if indicated -Referral to specialized care (i.e., orthopedic and spinal surgery) where indicated
Polypharmacy	-Thorough review of dose, duration, timing and indications for each medication -Search for indicated medications that have been omitted in prescribing (to avoid under-prescribing) -Review of over the counter and infrequently used medications -Assessment of cannabis product use, as well as frequency and route -Pharmacist review, assessment of medication, dose, correct usage by patient	-Education -Tapering and stopping medications no longer indicated -Re-consideration of prescriptions in which there are drug-drug and/or drug-disease interactions -Adjusting dosages based on renal and hepatic function -Prescribing indicated medications that were previously omitted -Blister packs/dosettes -Medication assistance or oversight when impaired cognition is present
Visual and hearing impairment	-Bedside hearing and vision assessment -Audiologist assessment -Optometrist and/or ophthalmologist assessment for glaucoma, cataracts, vitreous opacities (associated with ATTR)	-Hearing aids and amplifiers -Vision aids -Medications and surgery when indicated
Sleep disorder	-Comprehensive history and review of past medical history -Consideration of co-existing medical comorbidities such as obstructive sleep apnea and cognitive disorders, such as Lewy Body Dementia which may present with sleep disorders -Screen for depression/anxiety	-Patient education around changes in sleep with aging -Sleep hygiene -Exercise prescribing -Minimize caffeine -Referral to Cognitive Behavioral Therapy -Referral for sleep study if indicated

*ATTR, transthyretin amyloidosis; PSA, prostate specific antigen*.

## Approach to ATTR-CM Recognition and Diagnosis

Considering the challenges in recognizing ATTR-CM, an approach suggested by current literature includes ATTR-CM screening in patients presenting with multiple manifestations of disease, such as patients presenting with heart failure and peripheral neuropathy, autonomic dysfunction, or spinal stenosis, for example (2–4, 14, 15). Another suggestion is screening patients with unexplained increased LV wall thickness on imaging whose demographic profile put them at risk for ATTR-CM, including their age and ethnicity (4). Many patients with ATTR-CM are initially recognized following screening cardiovascular investigations for evaluation of heart failure or arrhythmia symptoms. Typically, this would include increased LV wall thickness with preserved biventricular chamber size on echocardiography or cardiovascular magnetic resonance imaging (CMR), and elevated cardiac biomarkers including troponin and B-type natriuretic peptide or amio-terminal pro-B-type natriuretic protein (BNP/NTproBNP) (2–4). Other common ATTR-CM findings on cardiovascular investigations are described in Figure 1. When cardiac amyloidosis is suspected based upon either clinical presentation or investigation findings, an important next step is to exclude AL (light chain) amyloidosis by testing for the presence of monoclonal protein in the serum and urine through electrophoresis and free light chain assay testing. The presence of monoclonal protein may suggest a plasma cell dyscrasia and trigger referral to a hematologist or oncologist for further testing (4). If AL amyloidosis is excluded, the recommended approach to non-invasive confirmation of ATTR-CM diagnosis is to perform technetium nuclear scintigraphy with bone-seeking radiotracer (including pyrophosphate, PYP and 3,3-diphosphono-1,2-propanodicarboxylic acid (DPD). If technetium nuclear scintigraphy is either unavailable or results are equivocal then cardiac biopsy should be performed. Once ATTR-CM is diagnosed, genetic testing should be performed to differentiate the wild-type from hereditary subtype (2–4).

## Care Considerations of Older ATTR-CM Patients

When considering the care needs of older patients with ATTR-CM, it is important to recognize that the experience of aging goes beyond a patient's age and is unique to each individual. The physiologic effects of aging impact individuals differently and are influenced by many factors including comorbidities, lifestyle and living conditions and psychological factors, among others (40). These factors can significantly influence how a patient responds to stressors such as chronic diseases and should be considered when providing care for ATTR-CM patients. The Geriatric 5M's summarize considerations related to older patient care and include: mind (cognition), mobility, medications, and matters most (individual care goals and preferences) (41).

### Mobility, Functional Capacity, and Living Situation

The clinical manifestations of ATTR, specifically the neurologic (including autonomic dysfunction) and musculoskeletal manifestations, can significantly impair a patient's mobility and functional capacity. Problems with balance, gait and the use of hands and upper extremities can significantly diminish a patient's independence and increase fall risk, among other complications associated with reduced mobility (30). These risks can be aggravated by cardiovascular manifestations such as orthostatic hypotension and heart failure. Patients may become dependent on mobility aids such as canes, walkers, or wheelchairs. Living environment and the level of caregiver support can significantly impact these limitations and risks. Assessment and treatment by occupational and physical therapists should be engaged for patients presenting with these limitations, and social work support may be required to coordinate additional assistance in the patient's home (i.e., home care) or transition to an assisted living facility when indicated.

### Polypharmacy

Aging is associated with the presence of multiple comorbidities, therefore addressing polypharmacy is an important aspect of the care of older adults with ATTR-CM (40). Polypharmacy is most often defined as the use of ≥5 daily medications, irrespective of the appropriateness of each medication (42). Prior studies have reported that nearly 50% of all individuals over 65 years have been prescribed medications that are no longer indicated (43). Polypharmacy has been reported to negatively impact mobility and was identified as an independent factor for falls leading to hip fractures in older patients (44). Multiple studies have identified a high prevalence of polypharmacy among heart failure patients, and an association with adverse outcomes (44–47). Polypharmacy may be particularly important and prevalent in patients with ATTR-CM, many of whom may be treated with commonly prescribed cardiovascular medications which are generally not well-tolerated by ATTR-CM patients, such as beta-blockers and vasodilators. These medications can contribute to exertional intolerance, fatigue, and hypotension in this population, and older patients are particularly susceptible to these adverse effects. Other adverse effects of polypharmacy include higher rates of malnutrition, increased urinary incontinence, which is more common in women over 70 years-of-age, increased risk of cognitive impairment (especially delirium) and a reduced ability to perform activities of daily living (44). These reports emphasize the importance of a multidisciplinary approach to the care of older adults to combat this risk, including a pharmacist and elder care specialist (48). Previous studies have demonstrated the effectiveness of a multidisciplinary approach to improve the overall quality of medication prescribing in older adults (44).

### Goals of Care

ATTR-CM is associated with a high burden of morbidity and mortality, even with the recent introduction of novel disease modifying therapies, and this is highly prevalent for patients with more advanced stage disease. Discussion with patients to ensure they understand how ATTR impacts them and their prognosis will help facilitate more productive discussions regarding management strategies that are most appropriate for each individual. Developing an understanding of older ATTR-CM patients' expectations and perspectives on their disease is very important for clinicians to inform all treatment decisions related to their care. For example, some older patients, particularly those with more advanced symptoms and/or significant limitations, may desire a less intensive approach to care that focuses on quality of life and symptom control rather than length of life. Some older patients may prefer to minimize appointments, investigations and testing, and use of medications that do not directly relieve or improve symptoms. Such goals of care discussions are an essential component of care delivery for many older patients with chronic diseases, and their approach and value is well-described in the heart failure literature (49, 50). These discussions often involve family members or other caregivers, and again may be informed by a multidisciplinary approach involving other specialist services such as geriatric medicine, palliative care and social work, among others. Such perspectives and considerations are often dynamic over time, and influenced by patients' symptoms, anticipated prognosis, and life circumstances, and may need to be revisited over time as these factors evolve. Palliative care specialists can contribute significantly to the management of older ATTR-CM patients, both with respect to symptom control and helping to educate patients and family or caregivers to determine the level of care that is most appropriate and meets the goals and values of the patient. Palliative care specialists are often consulted only at the very end-of-life, but can be a valuable part of developing a longer term care plan well in advance of this stage (40), making them an important member of a multidisciplinary ATTR care team for older patients (51).

### Genetic Testing

Multiple consensus guideline documents recommend performance of genetic testing for patients with confirmed ATTR-CM to confirm subtype (3, 4, 14). The rationale for this recommendation, even in older patients who are more likely to have ATTRwt, is that the results of genetic testing are important for determining eligibility for novel ATTR disease modifying therapies, assessing prognosis and the risk for extra-cardiac involvement, and determining the need to screen family members. This rationale should be carefully explained so that patients are comfortable with proceeding with genetic testing and are informed about the implications for them and their family. Older patients may have questions and concerns about the impact of the results on their children and other family members, including potential implications for life insurance eligibility or other financial and future-planning considerations of having an inherited disease. Referral to a genetics counselor is recommended to explore these issues in depth with a patient and their family (51).

## Frailty Assessment

Frailty has been variably defined but is generally accepted to be a multidimensional clinical syndrome that reflects a state of decreased physiological reserve and vulnerability related to aging (52, 53). Frailty manifests as an individual's impaired ability to recover from stressors, and while age represents an important risk factor, it is not a prerequisite for, nor the only factor associated with the development of frailty, which can include comorbidities and psychosocial factors, among others (54). Frailty has proven to be a powerful predictor of adverse clinical outcomes across a broad range of disease states and therapeutic interventions (55). This is particularly true in cardiovascular medicine (56, 57), where frailty has demonstrated prognostic significance for patients with heart failure (58), those undergoing transcatheter aortic valve replacement (TAVR) (52), coronary artery bypass grafting (CABG) (59), and others (60, 61). Frailty is highly prevalent in the general community and its incidence is rising with the aging population. A recent report estimated that 10% of community-dwelling older adults are frail and that these patients have a two-fold increased risk for mortality if they have a cardiovascular disease (53). Other reports have described that 25–50% of adults over 85 years-of-age are estimated to be frail with an increased vulnerability to sudden health changes, risk of falls, disability, the need transition to long-term care facilities, and other adverse outcomes (54).

Given the older age demographic of ATTR-CM patients, frailty may also be an important predictor of outcomes in this population. Currently limited evidence is available examining the prevalence and prognostic significance of frailty in ATTR-CM. A recent single-center report found that 39% of ATTR-CM patients followed met diagnostic criteria for frailty using the Clinical Frailty Scale (CFS), a validated tool that stages frailty according to a 9-point scale (with higher values indicating greater frailty) based upon semi-quantitative clinical evaluation (62). Frailty was also associated with all-cause mortality independent of ATTR-CM disease stage (62). Another report examining frailty phenotype in ATTRwt patients found a prevalence of frailty of 50% and 33% using the physical frailty phenotype and Short Emergency Geriatric Assessment questionnaire, respectively (38). Balance disorders and poor mobility were associated with duration of amyloid disease (38). The severity of cardiac amyloidosis was also associated with multiple frailty domains independent of age (38). Frailty evaluation has become an important component of evaluation and risk prediction prior to cardiovascular therapeutic interventions such as TAVR and CABG, and it has been speculated that assessing frailty may be similarly predictive of response to novel disease modifying therapy for ATTR-CM patients (62, 63). This may be particularly pertinent given the very high cost of these agents (64, 65). Despite a lack of evidence regarding the predictive value of frailty assessment to determine response to disease modifying ATTR therapy, a recent consensus guideline describing the management and follow-up of ATTR-CM discussed its potential value and recommended further research to better understand how frailty assessment can be optimally utilized for ATTR-CM patient care (63).

One of the challenges of implementing frailty assessment into clinical practice includes the multiple validated indices available, and lack of consensus on which to use. Some indices, such as the CFS, are based upon clinical evaluation and judgement and are quick and easy to incorporate, while others have multiple domains and require specialized equipment (such as a handheld dynamometer to measure grip strength) and specialized expertise to perform, and may be quite time consuming (40, 66, 67). Many frailty tools focus on core physical domains that define frailty phenotypes such as weakness, slowness, reduced activity, fatigue and weight loss, while others include assessment of cognition and mood (53). If frailty assessment is to be routinely incorporated into the evaluation of older ATTR-CM patients, then a standardized approach would facilitate optimal use, and further research is needed to understand which frailty assessment tool is most appropriate for patients with ATTR-CM.

**Figure 2:**
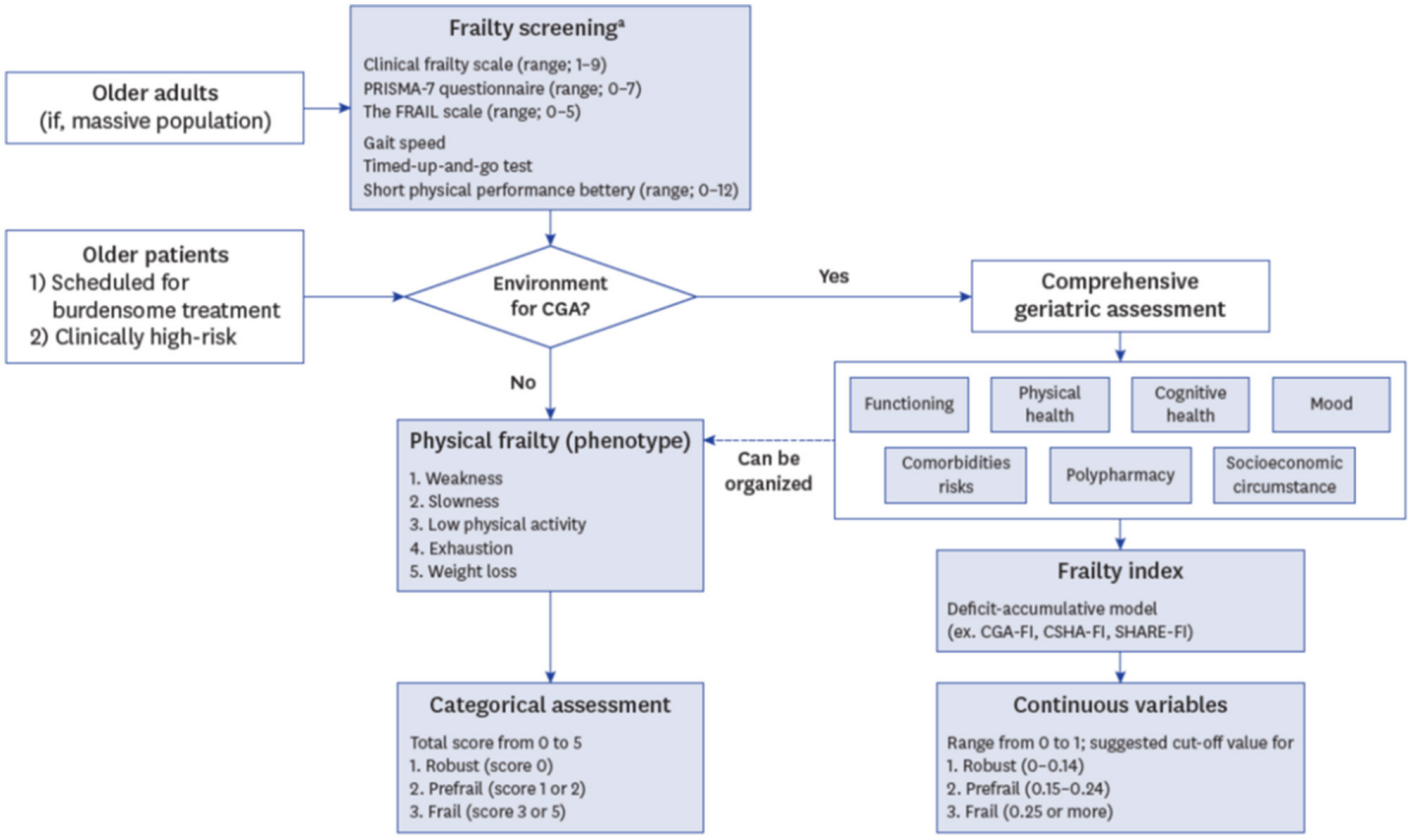
Schematic of incorporation of frailty assessment and comprehensive geriatric assessment (CGA) into clinical practice. FI, frailty index; CSHA, Canadian Study of Health and Aging; SHARE, Survey of Health; Aging and Retirement in Europe. Lee et al., (66). ^a^, Frailty screening can be omitted due to clinician's decision if comprehensive geriatric assessment is more necessary or available.

Routine frailty assessment should be viewed as a screening tool used to recognize and stage frailty. These screening measures often lack prescriptive elements however identification of frailty should then prompt further evaluation designed to improve frailty and reduce its associated risks. Comprehensive geriatric assessment (CGA), the gold standard for identifying frailty (54), is a multidimensional interdisciplinary process that is designed to identify potentially modifiable components of the frailty syndrome to maximize physical, psychological, and social health of elderly patients (66). The CGA incorporates a multidisciplinary approach to care and includes information on activities of daily living, cognitive and nutritional status, comorbidities, and medications to serve as a diagnostic and prognostic assessment tool for clinicians. A schematic approach to incorporation of frailty assessment and CGA into clinical practice is shown in Figure 2 (66). The CGA has demonstrated efficacy for the management of cancer patients, helping to predict the risk of toxicity to cancer therapy and decline in quality of life after treatment initiation (68). For cardiovascular clinicians caring for older ATTR-CM patients, frailty screening may then prompt referral of patients identified as frail for further multidisciplinary care and collaboration with geriatricians and elder care specialists, who can provide the expertise needed to identify and treat modifiable risk factors for frailty and otherwise help to manage these complex patients and aid in clinical decision making the ATTR-CM. As field awaits further research and evidence-based recommendations regarding optimal approaches for frailty screening, assessment and optimization for ATTR-CM patients, a suggested approach for cardiovascular clinicians is to screen for frailty using the CFS, which is a simple and easy to use and interpret index of frailty severity. Patients identified as frail with a CFS score of ≥5 could then be referred on to a geriatrician or elder care specialist for further assessment and management using the CGA (62).

## Management Considerations

The management of ATTR-CM generally involves two fundamental objectives; the first is to manage the symptoms and complications of disease, and the second is to modify disease course and attenuate further progression through the use ATTR-specific therapies (4, 63), Management considerations for older ATTR-CM patient with respect to both objectives will be discussed below.

### Symptom and Complication Management

Management of the symptoms and complications of ATTR-CM involves treatments such as diuretic therapy for heart failure and volume overload, anticoagulation for thromboembolic complication prophylaxis (namely stroke) for patients with atrial fibrillation, and management of arrhythmias, such as permanent pacemaker implantation for patients with symptomatic bradycardia or high-grade heart block (3, 4). In addition to concerns regarding polypharmacy, older adults with ATTR-CM may have impaired clearance and bioavailability of medications thereby impacting the optimal dose administered, and consultation with a pharmacist is of value in this setting. Atrial fibrillation is highly prevalent in ATTR-CM and its management can provide unique challenges in this population, particularly in older patients (23). Medications commonly used for heart rate-control are often poorly tolerated, such as beta-blockers (69), or contraindicated, such as calcium channel blockers or digoxin, which have been associated with increased risk of local (myocardial tissue) toxicity and heart block (70–72). Low dose amiodarone has been advocated for patients intolerant of beta-blockers who require therapy for atrial fibrillation (23). The role of implantable cardioverter-defibrillators (ICDs) in ATTR-CM remains controversial (5). A survival benefit from ICD therapy has not been proven, however among ATTR-CM patients with an ICD, appropriate shocks/therapies have been reported (73). Previous guidelines have suggested there is limited utility of ICDs for primary prevention of sudden cardiac death (4), owing to uncertainty regarding the mode of cardiac death in ATTR-CM patients, which can include ventricular tachyarrhythmias, bradyarrhythmias, or electrical mechanical dissociation (pulseless electrical activity) (74). Secondary prevention indications for ICD implantation are generally endorsed (4). An increasingly recognized complication of ATTR-CM that is prevalent among older patients, especially those with ATTRwt, is aortic stenosis. Older patients with known or undiagnosed ATTR-CM represent an increasing proportion of referrals for TAVR consideration, resulting in a greater focus on screening of older aortic stenosis patients for concurrent ATTR-CM. The recently published RAISE score aims to improve clinical recognition of ATTR-CM among patients with aortic stenosis by providing a scoring system based on the presence of the following features; carpal tunnel syndrome, right bundle branch block, age >85 years, high sensitivity troponin-T value >20 ng/L, interventricular septal wall thickness ≥18 mm, E/A ratio >1.4, and Sokolow index <1.9 mV, with a reported area under the receiver-operating characteristic curve of 0.86 (*p* < 0.001) (28). While there has been concern that ATTR-CM patients may represent a higher risk population for adverse outcomes following TAVR, recent evidence suggests select ATTR-CM patients with aortic stenosis derive survival benefit from TAVR compared with medical therapy alone (28). While further research is needed to optimize the approach to risk stratification for ATTR-CM patients referred for TAVR, this report suggests that current approaches used for selection of non-ATTR-CM patients for TAVR may also be effective for ATTR-CM patients. Of note, frailty assessment is an important and recommended component of evaluation of patients referred for TAVR, with geriatricians and elder care specialists recognized as important members of multidisciplinary TAVR programs. Considering the complexities that the management of ATTR-CM patients presents, close collaboration with electrophysiologists and TAVR program members is recommended. Other extracardiac manifestations described above such as neurologic, autonomic dysfunction, gastrointestinal and musculoskeletal further speaks to the importance of a multidisciplinary team approach to management (51).

### Disease Modifying Therapies

Recent years have witnessed tremendous advancements in the treatment of ATTR. In a short period of time, medical management of ATTR has evolved from predominantly symptom control with limited use of off-label therapies with uncertain efficacy to approved ATTR-specific medical therapies proven in randomized placebo controlled clinical trials to modify disease progression (75). Tafamidis is a novel oral TTR stabilizer that binds to the TTR tetramer and reduces its dissociation (76), which is considered to be the primary pathologic step leading to aggregation of TTR fragments into amyloid plaques and their deposition in organ tissue (13). Tafamidis is presently the only approved medical therapy for treatment of ATTR-CM. Two other agents, inotersen (an antisense oligonucleotide) and patisiran (a micro-ribonucleic acid inhibitor) are injectable therapies classified as TTR gene silencers that are approved for the treatment of ATTRh polyneuropathy only, and act by reducing hepatic TTR production (77, 78). Multiple other ATTR disease modifying therapies are in various stages of development, suggesting more treatment options will become available in the years to come (75). This presents at once an exciting, dynamic and rapidly evolving landscape for clinicians managing an already complex multisystem disease. Such complexities may be compounded for older ATTR-CM patients. And while some patients with ATTR may be eligible for solid organ transplant, such as liver transplant as a disease modifying treatment for ATTRh or heart transplant for patients with end-stage heart failure (79, 80), these therapies are generally not available to older patients because of their relatively higher surgical risk, and in fact are becoming less frequently performed owing to improvements in disease modifying medical therapies (4).

The Tafamidis Treatment for Patients with Transthyretin Amyloid Cardiomyopathy (ATTR-ACT) clinical trial demonstrated an overall reduction in mortality and cardiovascular hospitalizations among ATTR-CM patients treated with tafamidis compared with placebo after 30 months of therapy (76). Tafamidis was also associated with a significantly lower rate of decline in 6-min walk test distance and quality of life, as measured using the Kansas City Cardiomyopathy Questionnaire Overall Summary (KCCQ-OS) score. The study cohort included predominantly older (median age 75 years), male (90%) ATTRwt (76%) patients. Of note, tafamidis efficacy was higher in patients with New York Heart Association functional class I-II symptoms compared to those with class III symptoms, suggesting that therapeutic response is superior in patients with earlier stage disease (76), a characteristic common to other infiltrative disorders. Also of note is that tafamidis is a very expensive medication, with a list price in the US of approximately $225,000 per upatient per year (64). Tafamidis is in fact the most expensive cardiovascular medication ever listed in the US, and prices in other countries are similarly high if not higher (64, 65). A recently published study examined the cost effectiveness of tafamidis in terms of quality-adjusted life years (QALY) gained using a simulation model calibrated to the results of the ATTR-ACT trial (76). The study reported an incremental cost-effectiveness ratio of $880,000/QALY gained with tafamidis treatment, well-above the $100,000/QALY gained ratio generally recognized as an acceptable cost-benefit threshold for new therapies. The study estimated that treating all eligible ATTR-CM patients in the US with tafamidis would increase annual health care costs by $32.3 billion (64).

These aspects of tafamidis therapy create important considerations for their use in older patients. Although tafamidis improves prognosis, as a TTR stabilizer it generally does not improve symptoms, and patients may still experience disease related morbidity and progression while on treatment. Furthermore, long-term outcomes for patient on tafamidis have not yet been reported. Communication of these aspects of tafamidis therapy to patients is very important so that they will have realistic expectations of the benefits and limitations of treatment in the context of their individual goals of care. Some older patients may wish to focus on therapies that improve symptoms rather than extending life. In many regions, eligibility criteria for tafamidis reflect those used in the ATTR-ACT clinical trial. The main exclusion criteria were >90 years-of-age, NYHA functional class IV symptoms and 6-min walk test distance <100 m, and advanced hepatic or renal dysfunction or malnutrition (defined as a modified body mass index <600) (76). Further research is needed to identify other markers that will predict a response to therapy so that this highly expensive medication is used appropriately in patients with a life-expectancy that will allow them to derive benefit. This is particularly relevant to older patients, who may have other comorbidities and conditions that significantly limit life-expectancy. It is notable that in the ATTR-ACT trial, the benefits of tafamidis therapy over placebo were not observed until patients had received treatment for approximately 18-months (76). Validated ATTR-CM disease staging systems have been published (81, 82), although they are used variably in clinical practice with respect to determining candidacy for tafamidis therapy, and also do not take into account other potentially important non-cardiovascular factors that may limit life-expectancy such as frailty. Development of a more comprehensive approach to predicting therapy response and determining eligibility may aid clinical decision making with respect to the use of ATTR disease modifying therapies in older patients, particularly with other agents in development that are likely to be similarly high priced should they ultimately be approved.

## Care Gaps and Future Directions

A number of important knowledge and evidence gaps remain regarding the management of older ATTR-CM patients. In particular, the role of frailty assessment, including the optimal frailty assessment tool and how best to use the results, should be a focus of research for referral centers. Frailty assessment may contribute to the management of older ATTR-CM patients both in regard to identifying and treating modifiable factors that contribute to frailty with the goal of improving it, as well as determining appropriateness of ATTR therapy based on knowledge of the patients' goals and preferences, and the likelihood of treatment efficacy. However, at present such theories remain unproven and therefore premature for implementation into routine clinical practice. Beyond frailty assessment, further research focused on the characteristics and outcomes of older patient ATTR-CM patients, in particular those with ATTRh, is needed as the incidence and prevalence of ATTR-CM increases, to better understand and meet their complex clinical care needs. Such research should include focus on goals of care considerations and patient reported outcomes. This will aid in further developing and refining care pathways to facilitate timely evaluation by specialists who may improve the care of ATTR-CM patients. While geriatricians and elder care specialists are particularly important in this population, other specialists will also play a valuable role including neurologists, hand and orthopedic surgeons, palliative care providers, occupational and physical therapists, social workers, and pharmacists, in addition to other cardiovascular medicine specialists such as electrophysiologists and valvular heart disease experts. Ultimately, a comprehensive geriatric referral and care guideline should be developed for health care providers to guide interventions in older adults diagnosed with ATTR-CM.

## Conclusions

The prevalence of ATTR-CM is likely to continue to rise with improvements in disease awareness and diagnostic approaches in the context of an aging population. Advancements in disease modifying therapy will improve survival. With a greater number of older ATTR-CM patients being diagnosed and living longer, the need for a patient-centered multidisciplinary care approach rises for this complex patient population. Many of the manifestations of this multisystem disease can exacerbate other age-related disorders. Collaboration and shared care with specialists who have expertise in managing challenges prevalent among older patients, including polypharmacy and goals of care assessment, has become essential for cardiovascular clinicians caring for ATTR-CM patients. Frailty assessment is anticipated to become increasingly important for this population, however further research is needed to determine how best to assess frailty and incorporate it into clinical care. Whether this plays a role in determining how expensive ATTR disease modifying therapies such as tafamidis are prescribed for older ATTR-CM patients warrants further research, especially with multiple other therapies currently under investigation.

## Author Contributions

BI, JM, and NF contributed to the conceptualization, literature review, writing, review, and approval of this manuscript. All authors contributed to the article and approved the submitted version.

## Conflict of Interest

NF has received research funding support and consulting and speaking honoraria from Pfizer, Alnylam, Akcea, Ionis and Eidos. The remaining authors declare that the research was conducted in the absence of any commercial or financial relationships that could be construed as a potential conflict of interest.

## Publisher's Note

All claims expressed in this article are solely those of the authors and do not necessarily represent those of their affiliated organizations, or those of the publisher, the editors and the reviewers. Any product that may be evaluated in this article, or claim that may be made by its manufacturer, is not guaranteed or endorsed by the publisher.
